# Significant Implications of *APOA1* Gene Sequence Variations and Its Protein Expression in Bladder Cancer

**DOI:** 10.3390/biomedicines9080938

**Published:** 2021-08-02

**Authors:** Javid A. Magray, Arshad A. Pandith, Iqbal Qasim, Muzzain Khateeb, Arif Hamid, Aabid Koul, Zafar A. Shah, Shahid M. Baba, Sheikh Mansoor, Wafa Charifi, Ajaz Ahmad, Mohammad S. Wani

**Affiliations:** 1 Department of Urology, SK Institute of Medical Sciences (SKIMS), Srinagar 190011, India; dr.javaid82@gmail.com (J.A.M.); abdulkhwaja1@gmail.com (M.K.); arifhamid10@gmail.com (A.H.); 2 Advanced Center for Human Genetics, SK Institute of Medical Sciences (SKIMS), Srinagar 190011, India; iqbalaiims@gmail.com (I.Q.); aabidmustafakoul@gmail.com (A.K.); bshhid007@gmail.com (S.M.B.); mansoorshafi21@gmail.com (S.M.); 3 Immunology and Molecular Medicine, SK Institute of Medical Sciences (SKIMS), Srinagar 190011, India; zaffaramin@gmail.com; 4 Cochin Institute, University of Paris, INSERM, U1016 Paris, France; wafa.charifi@hotmail.fr; 5 Department of Clinical Pharmacy, College of Pharmacy, King Saud University, Riyadh 11451, Saudi Arabia; ajukash@gmail.com

**Keywords:** bladder cancer, *APOA1*, genotype, allele, protein expression, haplotype

## Abstract

Apolipoprotein A1 (*APOA1*) is a potential biomarker because of its variable concentration in different types of cancers. The current study is the first of its kind to evaluate the association between the *APOA1* genotypes of −75 G/A and +83 C/T in tandem with the *APOA1* protein expression in urine samples to find out the risk and potential relationship for differentially expressed urinary proteins and *APOA1* genotypes. The study included 108 cases of bladder tumors and 150 healthy controls that were frequency matched to cases with respect to age, sex, and smoking status. Genotyping was performed using PCR-RFLP and the urinary expression of the *APOA1* protein was done using ELISA. Bladder tumor cases were significantly associated with the *APOA1* −75 AA genotype (*p* < 0.05), while the *APOA1* +83 C/T heterozygotes showed an association with cases (*p* < 0.05). The overall distribution of the different haplotypes showed a marked difference between the cases and controls in GT when compared with the wild type GC (*p* < 0.03). Bladder tumor cases that carried the variant genotype *APOA1* −75AA were found more (70.0%) with a higher expression (≥20 ng/mL)of the *APOA1* urinary protein and differed significantly against wild type GG (*p* = 0.03). Again, in low grade bladder tumors, urinary *APOA1* protein was exhibited significantly more (52.4% vs. 15.4% high grade) with a higher expression (≥20 ng), while high grade tumor cases (84.6% vs. 47.5% low grade) showed a lower *APOA1* expression (<20 ng/mL) (O.R = 6.08, *p* = 0.002). A strong association was observed between *APOA1* −75G/A and risk for bladder tumor and its relation to urinary protein expression, which substantiates its possible role as a marker for the risk assessment of the disease and as a promising diagnostic marker for different grades of malignant bladder tumors.

## 1. Introduction

Bladder cancer is the ninth most common cancer worldwide and accounts for 3.9% of all cancer cases [1]. It is a cancer of the environment and age where the incidence rates increase with age, peaking in the eighth decade of life, and a strong association has been documented between environmental toxins, smoking, and urothelial cancer formation [2,3,4]. Apart from these risk factors, there are circumstances where the disease develops without or with very limited exposure to the risk factors. This suggests that susceptibility for many genes, in particular polymorphic variations, may play a role in the etiology of bladder cancer. The incidence rate of bladder cancer has been rising over the last 60 to 70 years, but the rate of increase has recently decreased significantly and in some geographic areas has leveled off [2,5].

Apolipoprotein A1 (APOA1) is the major apoprotein constituent of high-density lipoprotein (HDL) that can play important roles in tumor invasion and metastasis [6,7]. Recent findings revealed the crucial roles of *ApoA*-1 in inflammation, tumor growth, angiogenesis, invasion, and metastasis [8,9,10]. The *APOA1* gene is located on 11q23-q24, and encodes ApoA1 [7,11]. APOA1 is a constitutive anti-inflammatory factor, and the decrease in the HDL-associated apoA1 level may be a signal of chronic inflammation progression. It has been shown that the *APOA1* concentration in blood is reduced in different types of cancer [12,13]. *APOA1* has been identified as a potential biomarker of ovarian [9], colorectal [14], hepatocellular [13], and pancreatic cancer [15]. Among the many polymorphic sequence variations present in the *APOA1* gene [16], −75 G>T transition [17] and +83 C>T in the first intron, are reported to influence the HDL and apoA1 protein concentration [18,19].

Recently, *APOA1* −75 G/A and +83 C/T have been observed to have strong relation to renal tumors [20], and their protein expressions have been demonstrated to be potential biomarkers in bladder cancer [21]. The quest for proteomic non-invasive biomarkers is continuing to identify disease biomarkers in biological fluids that can be measured relatively inexpensively for the early diagnosis of disease. As urine is directly exposed through the bladder epithelium, it provides an attractive alternative to blood plasma as a potential source of disease biomarkers for bladder cancer. Although lipoproteins are believed to likely indirectly promote tumor survival or increase tumor angiogenesis, the relationship between the changes in the lipoprotein levels, *apoA1* with bladder cancer progression, and disparity in the lipoprotein-related proteome between bladder tissue and urine is still uncertain, and necessitates further investigation in order to understand its potential as a biomarker. Studies conducted recently have authenticated that *APOA1* (major component of HDL-C) is a promising prognostic biomarker for various malignancies [8,22,23]. Furthermore, some reports have substantiated that a higher expression of *APOA1* is significantly related to a better prognosis in nasopharyngeal and lung cancer [24,25]. As bladder cancer is the second most common urological cancer, characterized by high recurrence rates and a poor prognosis, there is a clear need to identify new tumor specific biomarkers for the early diagnosis and accurate classification of bladder tumors in order to make an appropriate therapeutic plan for cancer patients. Keeping in mind the plausible role of *APOA1* in various malignancies, a combined genetic and proteomic analysis of *APOA1* is hypothesized to help observe the predisposition to bladder cancer and may aid in the clinical diagnostic stratification of bladder cancer. To test this hypothesis, for the first time, to the best of our knowledge, a prospective hospital-based case-control study was undertaken to analyze the association of the *APOA1* −75 G/A and +83 C/T genotypes with predisposition to bladder cancer. In addition, we investigated the *APOA1* protein expression in the urine samples of patients with bladder cancer in order to find the potential relationship between differentially expressed urinary proteins and variations in the *APOA1* genotypes.

## 2. Material and Methods

### 2.1. Study Subjects

The present case-control study was conducted at the Department of Urology, in collaboration with the Department of Immunology and Molecular Medicine and Advanced Center for Human Genetics, Sheri-I-Kashmir Institute of Medical Sciences (SKIMS), North India. For the polymorphic analysis of *APOA1*, a total of 258 subjects were included in the study, comprising of 108 bladder tumor patients and 150 cancer free healthy controls. First, 5 mL blood from each subject was collected and stored at −20° for further analysis. The case and control subjects were recruited from the same hospital and all came from the same geographic area and had the same ethnic background. Various diagnostic approaches were employed for the diagnosis of bladder tumors, including cystoscopy (CPE), ultrasonography (USG), and CT scans. Upon CPE examination, most of the bladder cancer cases were papillary, cauliflower, and flat type, followed by sessile and solid type. Hematuria, a hallmark of bladder tumors, was seen in 62% of patients. All of the cases of bladder cancer included were new (de nova) and had been treated for the first time in the Department of Urology (SKIMS).

In addition, an overnight 12-h urine sample from each patient with a bladder tumor was collected in a container and after proper shaking, 50 mL of the sample was stored in a deep freezer (−20 °C) for the expression analysis of *APOA1* by ELISA. The study was approved by the Ethical Committee of the Institute (SKIMS Study ref: Protocol 81/2013). Both the patients with bladder cancer and the healthy controls were included after written informed consent was obtained. Only those patients who had histopathologically proven bladder tumors were included in the study as cases. Clinical parameters were recorded according to the proforma given, and for history, special reference was given to any family history of tumors and any hereditary diseases. The subjects were considered as never smokers only if, until the day of sample collection, they had not used tobacco, and were considered current smokers if they were smoking presently or had quit smoking 6 months prior or less before sample collection.

### 2.2. Extraction of GenomicDNA

The blood and tumor tissues of the bladder cancer patients and healthy controls were subjected to the extraction of genomic DNA using the phenol chloroform method, as well as a DNA extraction kit (Zymo Research Corporation, Irvine, CA, USA).

### 2.3. Polymerase Chain Reaction for the Amplification of APOA1 −75 G/A and +83 C/T

To amplify the *APOA1* −75 G/A and +83 C/T polymorphic regions, we used genomic DNA:100–250 ng/mL, 1× PCR buffer: 100 mM Tris–HCl, pH 8.3; 500 mM KCl; 20 mM MgCl2; DNTP (Sigma–Aldrich, St. Louis, MO, USA): 10 mM dATP; 10 mM dCTP; 10 mM dGTP; 10 mMdTTP, primers (Sigma–Aldrich, St. Louis, MO, USA), and *Taq DNA polymerase* 5 U/μL (Biotools, Madrid, Spain). The primer pair used amplified the 433 bp amplicon covering both of the SNPs of the interest within the *APOA1* region with the forward primer 5′-AGGG ACAGAGCTG ATCCTTGAACTCTT AAG-3′ and reverse primer 5′-TTAGGGGACACCTAGCCCTCAGGAAGAGCA-3′. The thermal conditions used were first a denaturation step at 94 °C for 5 min, 35 cycles of denaturation at 94 °C, annealing at 58 °C, and extension at 72 °C for 30 s, followed by a final extension cycle at 72 °C for 5 min.

### 2.4. Polymerase Chain Reaction-Restriction Fragment Length Polymorphism (PCR-RFLP)

PCR-RFLP analysis was employed to differentiate different genotypes of the *APOA1* −75 G/A and +83 C/T polymorphic sequence variants. *MspI*, a restriction endonuclease enzyme (New England Biolabs, NEB, England), was used to digest the 433 bp amplicon overnight. The amplified product was subjected to electrophoresis in a 3% agarose gel, followed by ethidium bromide staining and ultraviolet illumination to identify the genotypes. The *MspI* restriction site at −75 bp (G allele) and +83 bp (C allele) generated fragments of 45, 66, 113, and 209 bp, while the loss of the restriction site at 75 bp (A allele) produced 45, 179, and 209 bp. Similarly, the loss of the restriction site at +83 bp (T allele) displayed 254 bp instead of two fragments of 45 and 209 bp. To ensure quality control, two independent observers randomly selected 5% of the samples from each group for genotyping in order to confirm the reproducibility of the results.

### 2.5. Expression of APOA1 in Urine of Bladder Tumor Patients by ELISA

The soluble urinary *APOA1* concentrations were measured with ELISA using standard source ELISA kit (Thermo Scientific, Waltham, MA, USA) urine samples from both the bladder tumor and control groups, which were centrifuged for 15 min at 1500 rpm, and the supernatants subsequently utilized for protein expression by ELISA.

ELISA was performed according to the methods recommended by the manufacturer. The standard was generated by plotting the curve obtained for each standard concentration and the results were interpreted using a curve-fitting statistical software package. If using curve fitting software, we plotted a four-parameter logistic curve fit. After the development of the colorimetric reaction, the OD readings were converted to nanograms per milliliter (ng/mL) on the basis of the standard curves obtained with *APOA1* standard preparation in the assay. Each sample was tested in duplicate. The *APOA1* concentrations were represented as mean ±SD.

### 2.6. Statistical Analysis

The cases and controls were compared using the chi-square test for categorical variables, such as sex and smoking status, and the demographic variables. A goodness-of-fit chi-square test was used to determine whether the polymorphisms were in Hardy–Weinberg equilibrium for the cases and controls. Odds ratios (OR) were used as the estimates for the relative risk, and 95% confidence intervals (CI) were calculated to estimate the association between certain genotypes or other related risk factors of bladder cancer.

## 3. Results

For *APOA1* −75 G/A and +83 C/T polymorphic sequence variations and urinary protein estimation, the cases included 90 (83.4%) males and 18 (16.6%) female patients, and the controls consisted of 123 (82%) males and 27 (18%) females. The cases and controls were closely frequency matched in terms of their age, gender, and smoking status. The median age for bladder tumor cases was estimated at 56.5 and 53.5 for the healthy controls. Of the total number of cases, 90 (86.1%) were smokers and the frequency was almost matched with the controls, being 115 (76.7%), depicted in Supplementary Table S1. Among the cases, 75 (69.4%) patients were above 50 years of age, and no significant age- or gender-related differences were observed between the groups (*p* > 0.05). Low grade bladder tumors were present in 88 (81.5%) patients. The pathological stages found in patients Ta, T1, and T2 were found in 30 (27.7%), 63 (58.3%), and 15 (13.8%), respectively.

### 3.1. Analysis of APOA1 −75 G/A Polymorphic Sequence Variation

In *APOA1* −75 G>A, the frequencies of the GG, GA, and AA genotypes among cases were 48 (44.4%), 40 (37.0%), and 20 (18.5%), while in the controls, the frequencies were 81 (54%), 60 (40%), and 9 (06%), respectively. When variant genotype AA was compared between the two groups, a higher frequency was observed in the tumor cases (18.5%) compared with the controls (6%), with an odds ratio (OR) of 3.7 (95%CI: 1.24–10.3), and the difference for the variant AA genotype among cases and controls was observed to have a statistically significant association (*p* = 0.001; Table 1). The distribution of the heterozygote (GA) genotype among the cases and controls was 37.0% vs. 40.0%, with an OR of 1.25 (C.I; 0.6–1.9) (*p* = 0.5). The cases had a higher frequency for the combined variant genotype (GA + AA; 55%), compared with the controls (46%), with an OR of 1.2 (C.I: 0.56–1.99, *p* > 0.05) (Table 1).The frequency of the variant ‘A’ allele observed in the cases and controls was found to be 37.1% vs. 26.0%, respectively, with an OR of 1.6 (C.I; 1.03–2.61), and the difference showed an association of the variant allele (A) for cases (*p* = 0.004; Table 1). No major difference in the frequency of either the wild or variant genotype was found when considering gender or patients with different smoking status compared with the control group (Table 2).

When the patients were classified as cohorts with respect to their different pathologies, variant genotypes GA + AA were found significantly more in low-grade compared with high grade bladder tumors, with a frequency of 75.0% vs. 25.0%, respectively, with an OR of 3.2 (*p* = 0.04; Table 2). Similarly, the variant genotypes showed an elevated trend, as the depth of invasion increased from pTa < pT1 < pT2 stages of the bladder tumors (43.7% < 53.9 % < 80%), but the GA + AA genotype frequency between the Ta and T2 stages (43.7% vs. 80%) differed significantly (*p* = 0.05; OR; 4.4; Table 2).

### 3.2. Analysis of APOA1 +83 C/T Polymorphic SequenceVariation

In *APOA1* +83 C/T, the frequencies of the CC, CT, and TT genotypes observed among bladder cases were 44 (40.3%), 54 (50.0%), and 10 (9.7%), while on the other hand, the healthy control frequencies were 84 (55%), 48 (33%), and 18 (12%), respectively. The heterozygote CT genotype was significantly higher for the cases (50.0%) than the controls (33.0%), with an OR of 2.1 (C.I; 1.07–3.97, *p* = 0.005). Interestingly, when the variant genotype TT was compared between the two groups, a higher frequency was observed in the healthy controls (12%) compared with the cases (9.7%), with an OR of 1.1 (CI: 0.54–3.8, *p* = 0.6). Although the cases had a higher frequency for the combined variant genotype (CT + TT) (59.7%) than the controls (45.0%), with an OR of 1.3 (C.I: 0.98–3.34), there was no significant difference between the two groups (*p* > 0.05). The frequency of the variant ‘T’ allele observed in the cases and controls was found to be 33.3% vs. 28%, respectively, with an OR of 1.3 (C.I: 0.53–1.40, *p* > 0.05).When classified further into groups, as depicted in Table 2, our study found a higher frequency of variant genotypes (CT + TT) in the age group ≥50 years (57.7% vs. 46.4%) in patients <50 years (*p* > 0.05). In the gender evaluation, females presented a significantly higher variant genotype (CT + TT) frequency in the tumor cases (66.7%) compared with the controls (30.0%), with an OR of 4.4 (CI:1.3–15.4) (*p* = 0.03). Again, when the patients were classified as cohorts with respect to their different pathologies, variant genotypes CT + TT were found more in the high-grade than the low grade bladder tumors, with a frequency of 62.1% vs. 50.0%, respectively (*p* > 0.05). Similarly, as found in the *APOA1* −75 G/A variant genotypes, *APOA1* +83 C/T also showed an elevated trend of CT + TT variant genotypes as the depth of invasion increased from pTa > pT1 > pT2 stages of the disease, although the difference was not observed to be significant (*p* > 0.05). The associations of the variant alleles with other clinico-pathologic characteristics are given in Table 2.

Haplotypic analysis was done to observe the pattern of linkage disequilibrium, and we found haplotypes with frequencies more than 5% between both the cases and controls. The haplotype with the highest frequency observed was the G/C haplotype, with frequencies of 34.2% in the cases and 27.4% in the controls, followed by G/T, with 2.8% and 9.4%, respectively. Table 3 shows the frequencies for the estimated two-marker haplotypes between the patients and controls. The overall distribution of the different haplotypes showed a marked difference between the cases and controls in variant GT when compared with wild type GC (*p* < 0.03), as depicted in Table 3.

### 3.3. Urinary Protein Expression of APOA1 and Potential Relationship with APOA1 −75 G/A and +83 C/T

The *APOA1* protein was further tested quantitatively by ELISA so as to measure its differential expression pattern in order to distinguish the patients with bladder cancer of varied pathologies. Furthermore, the quantitative differential pattern of *APOA1* was analyzed with respect to the different genotypes/alleles of *APOA1* −75 G/A and +83 C/T. Once the calibration curves were proven to be analytically optimal, *APOA1* was measured in the urine samples of the bladder tumor patients using ELISA, according to the manufacturer’s protocol. The optimal cutoff for the bladder cancer cases was 20.0 ng/mL with an AUC of 0.889 (95% CI 0.758–0.998), and optimal cutoff for the controls was taken as 18.22 ng/mL, with an AUC of 0.912 (95% CI0.872–0.996).

**Figure 1:**
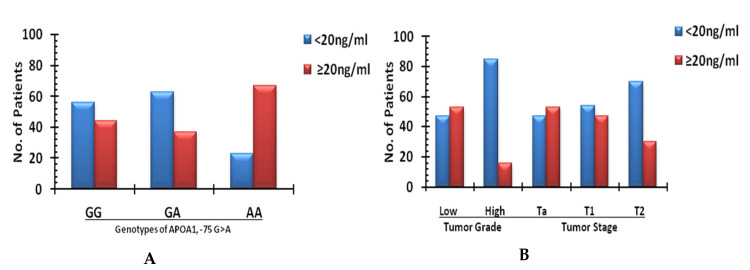
(**A**,**B**): Different concentrations of *APOA1* urinary protein with (**A**) genotypes of *APOA1* −75 G/A and (**B**) with different grades and stages of the patients with bladder cancer.

In *APOA1* −75 G/A, the protein levels of *APOA1* showed a higher distribution of wild type genotype GG, 60.4% for <20 ng/mL vs.39.5% in the protein concentration of ≥20 ng/mL. In contrast, the variant genotype AA frequency was observed to be lower, with 30.0% in <20 ng/mL, and a higher frequency of 70.0% in the ≥20 ng/mL *APOA1* urinary protein levels. The difference between the wild type GG and variant genotypes AA of *APOA1* −75 G/A was observed to be associated significantly with an OR of 1.5 (95% C.I; 0.34–4.59: *p* = 0.03; Table 4), and furthermore, Figure 1A shows the graphic representation of the significant distribution of the genotypes of *APOA1* −75 G/A among the different concentrations of the *APOA1* urinary protein. *APOA1* +83 C/T did not relate significantly to urinary *APOA1* concentration, as both the higher and lower protein levels were comparable among the genotypes (*p* < 0.05).

A higher expression of *APOA1* (≥20 ng/mL) was observed in low grade bladder tumor cases compared with the high grade ones (52.5% vs. 15.4%, respectively), and the difference in expression between the two groups was significant (*p* = 0.002) with O.R 6.08 (95%C.I = 1.24–29.8; Table 4).Among the different stages of bladder cancer cases, the expression levels of *APOA1* at <20 ng/mL were slightly less in the Ta stage (47.6%) compared with those with ≥20 ng/mL levels (52.4%). In comparison, the T1 stage had more (52.3%) cases with an *APOA1* concentration <20 ng/mL than the cases with ≥20 ng/mL levels (47.7%). For the T2 stage, 70% of cases presented with a lower *APOA1* concentration (<20 ng/mL) compared to 30.0% of cases with a higher concentration (≥20 ng/mL), with OR 2.3 when compared with Ta stage (*p* = 0.2). We observed a decreased trend in the *APOA1* levels (<20 ng/mL) with enhanced tumor stages (stage Ta to T1 and T2). Figure 1B shows the different concentration of the *APOA1* urinary protein with different grades and stages for the bladder tumor cases. Details about the relationships of the different parameters of bladder cancer cases with urinary *APOA1* are shown in Supplementary Table S2. A decreased *APOA1* concentration (<20 ng/mL) was presented in36 cases (58.1%) of smokers compared with 7 (70%) cases of non-smokers, while 26 (41.9%) smokers had an increased expression (≥20 ng/mL). The difference in the differential expression of *APOA1* among the smokers vs. non-smokers was statistically insignificant (*p* = 0.09). A significant association between the different patterns for *APOA1* expression was observed among gender, with a higher number of males (36; 50%) presenting with a lower expression (<20 ng/mL), while a higher expression (≥20 ng/mL) of *APOA1* was seen more in females (9; 75%) compared with 24 (33.4%) cases for males, with an OR of 4.5 (C.I: 1.10–18.34: *p* = 0.02). The age group classification was not associated with either a lower or higher *APOA1* concentration.

We stratified the various clinic-pathological parameters with respect to the distribution of different genotypes of *APOA1* −75 G/A and +83 C/T, so as to analyze their association with the protein levels of the *APOA1* gene, but no association was observed (Supplementary Table S2).In the case of smokers, a decreased *APOA1* (<20 ng/mL) was observed to be relatively high for cases in both variants of *APOA1*, but were not significantly associated (*p* > 0.05). Likewise, the distribution of the variant genotypes of *APOA1* −75 AA and +83 TT also showed an increased expression of the *APOA1* (≥20 ng/mL) protein for various pathological grades, but did not achieve statistical significance (*p* > 0.05).

## 4. Discussion

*APOA1*, a protein encoded by the *APOA1* gene [7,11], has a specific role in lipid metabolism. Various genetic variants of the *APOA1* gene are known, including *APOA1*, 75 G/A, and +83 C/T. It has been shown that the *APOA1* concentration in blood is reduced in different types of cancer [12,13]. *APOA1* has been identified as a potential biomarker of ovarian cancer [9], colorectal cancer [14], and pancreatic cancer [15]. Although there is no doubt that most polymorphisms are functionally neutral, some affect the regulation of the gene expression or the function of the coded protein [26]. Recent findings revealed the crucial roles of *APOA1* in inflammation, tumor growth, angiogenesis, invasion, and metastasis [8,9].

A prospective hospital-based case-control study was conducted to evaluate the association of the *APOA1* −75 G/A and +83 C/T genotypes with predisposition to bladder cancer. Our study is the first one to identify the *APOA1* protein expression in the urine samples of patients with bladder cancer, and to find out the potential relationship between differentially expressed urinary proteins with different sequence variants of in the *APOA1* −75 G/A and +83 C/T genotypes.

The frequency of the variant genotype *APOA1* −75 AA was observed to be higher in the cases than in the controls (18.5 % vs. 6%, respectively), and conferred a 3.7-fold risk to bladder tumor development (*p* = 0.001). This report clearly demonstrates a significant association of the *APOA1* −75 G/A genotype with the susceptibility of subjects to the development of bladder tumors in our population. Likewise, the frequency of the *APOA1* −75 variant A allele was found to confer a significant risk for bladder tumors compared with the controls (3.1% vs.26%, respectively; *p* = 0.004). A similar scenario was published earlier (20) in China, where *APOA1* −75 G>A was shown to be associated with risk for the development of renal cell carcinoma. In agreement with this study, the findings in our study corroborated the bladder tumor risk in terms of both the genotype *APOA1* −75 AA and the overall risk, with the *APOA1* −75, for the A allele, with a comparative frequency of GG, 50.0%; GA, 30.6%; and AA, 19.4% (20) vs. 44.4%, 37.0%, and 18.5% from our study. Similarly, the allele frequency showed a concordant relation between the work of Zhi Hong et al. (2015) and our study, as follows: G allele 0.65 vs. 0.64, and A allele 0.34 vs. 0.37 [20], respectively.

The *APOA1* +83 C/T SNP showed an inverse relation with respect to the risk of bladder tumor. The variant homozygous genotype *APOA1* TT presented with a comparable frequency in the controls and cases (12% vs. 9.7%, respectively, *p* > 0.05). On the other hand, the *APOA1* +83 heterozygous genotype CT frequency was seen more in the cases compared with the controls (50% vs. 33%, respectively), and showed a significant association to confer more than a two-fold risk for bladder cancer. This finding was in total contradiction to the study by Zhi Hong et al. (2015) [20], where the distribution of all three genotypes was nearly uniform and was not associated with renal cell cancer. The discrepancy could possibly be attributed to the nature and biology of the two different cancers under investigation. Furthermore, the allele frequencies between two studies showed some semblance with the C allele frequency in our study (0.66 vs. 0.77, respectively) and the T allele (0.28 vs. 0.22, respectively). As in our study, the ensuing report with the *APOA1* −75 G/A was found to be associated with gallstone disease [27]. The *APOA1* −75 G/A and +83 C/T genotypes were also associated with susceptibility to breast cancer and lymph node metastases occurrence, respectively [28]. The latter report seems to be in complete accordance with our study, with only the exception that we found being the association of *APOA1* +83 C/T in bladder tumor cases with a heterozygous condition (+83 CT). Hamrita et al. (2011) evaluated the role of functional *APOA1* polymorphisms (75 G/A and 83 C/T) as genetic markers for breast cancer susceptibility [28], and found no association between the 83 C/T genetic variation in the *APOA1* gene and the risk of breast cancer occurrence. The presence of the *APOA1* +83 T allele appeared, however, to be associated with an increased risk of lymph node metastasis, but this same finding is refuted in our report. Similarly, another study found *APOA1* polymorphisms (75 G/A and +83 C/T) as a risk for myocardial infarction in a North Indian population [19].

The results in our report seem to indicate that *APOA1* −75 G/A and +83 C/T could markers for risk assessment in bladder tumor, as the results from the individual studies from different regions have come up with similar findings in different diseases, including cancer. This is further substantiated by functional studies that have shown *APOA1* to play important roles in tumor growth, angiogenesis, invasion, and metastasis [8,9,10].

When the patients were stratified as cohorts with respect to the different bladder tumor pathologies, combined genotypes of *APOA1* −75 and GA + AA (75.0%) were associated with high grade bladder tumors, conferring a three-fold increased risk (*p* = 0.04). With regard to tumor stage, a higher trend was seen with the variant genotypes, as the depth of invasion increased from low to high stages for the tumors and the distribution of the variant genotype in T2 stage was nearly significant (*p* = 0.05). It is clear that *APOA1* derangement at a genetic level is mainly involved in late stage bladder tumors. This is substantiated by a study that revealed an overall protective ability of HDL, specifically *APOA1*, to induce tumor suppression through both innate and adaptive immune processes in multiple animal tumor models [29,30], which demonstrated that HDL’s anti-inflammatory properties were conferred, in part, through HDL-micro-RNA (miR)-223 delivery and the translational repression of ICAM-1 in endothelial cells. However, the miR221/222 cluster increases the aggressiveness of tumor sinepithelial cancers through the repression of tumor suppressors and the induction of cell motility [31]. Furthermore, *APOA1* allelic variety may have an impact on angiogenesis [32].

The impact of a single polymorphic variation is unlikely to cause an effect in the study of multifaceted diseases, and the combination of various genetic variants in the network of the same loci strengthens their impact and improves their prognostic influence on complex diseases [33,34,35]. To substantiate whether *APOA1* −75 G/A and +83 C/T polymorphic variants could confer a synergistic effect in the development or progression of bladder cancer, we explored their cumulative impact so as to observe their predisposition to the disease. The most frequently implicated haplotypes found were G/T when compared with wild type G/C haplotypes, and this data showed the haplotypes at two RFLP sites as more informative, and showed a significant association together to confer a risk for the predisposition of bladder cancer. A similar observation was unraveled by Kamboh et al. (1996) [36], who used haplotype analysis, combining both restriction sites, as they were more informative with almost twice the amount of phenotypic variation in the plasma *APOA1* as opposed to a single restriction site. Our report points out that haplotype analysis in the 5′ promoter region of the *APOA*l gene is very valuable for revealing the functional importance of this gene in bladder cancer.

In this study, the *APOA1* protein levels were measured and a classification was done according to the optimal cutoff values, which were taken as a measure of the mean in the control urine samples (18.22 ng/mL) and in the bladder tumor cases (20 ng/mL). The different cut off *ApoA*-1 levels were also correlated with varied distribution of *APOA1* genotypes and alleles. Lower levels of the *ApoA*-1 urinary protein showed a significant association with high grade tumors (<20 ng; 84.6% vs. 15.4%), while low grade tumors were related to a six-fold higher expression (≥20 ng). Among the different stages of bladder cancer, *ApoA*-1 showeda lower expression (<20 ng/mL) with each subsequent higher stage, namely Ta (47.6%), T1 (52.3%), and T2 (70%). On the other hand, the higher expression (≥20 ng) showed a progressive decline, as the depth of invasion increased from Ta, T1, and T2 (52.4 %, <47.7%, and <30.0%, respectively). The results from our study seem to associate the *ApoA*-1 expression with a lower intensity of bladder cancer, revealing a protective effect. Likewise, Xiao-Lin et al. (2015) found the increased *APOA*-I concentration significantly related to the improved prognosis, with respect to nasopharyngeal cancer [37]. The findings of our study are, however, in stark contrast with the one conducted by Hongjie et al. (2015), who showed elevated *APOA1* urinary protein levels associated with a higher stage disease [20]. The discrepancy of the results could be possibly due to the different set of cancers under investigation, as Hongjie et al. (2015) [20] studied the *APOA1* levels in renal cell cancer, and their cut off values for *APOA1* were quite high and different from our study. As bladder cancer may proceed with inflammation of the epithelium, our results corroborate the fact that *APOA*l is well-known as a negative marker of inflammation; its concentration decreased by more than 25% during inflammation. *APOA1* is a constitutive anti-inflammatory factor, and the decrease in the HDL-associated *APOA1* level may be a signal of chronic inflammation progression. The fact that a marked reduction in *APOA1* levels occur during inflammation corroborates well with our findings, where we found a definite correlation between a lower expression of *APOA1* with higher bladder tumor grades and stages. It has further been substantiated that the *APOA1* protein concentration in blood is reduced in different types of cancer, like ovarian cancer and hepatocellular cancer [12,13]. However, several reports have come up with controversial observations, wherein up-regulation has been shown in a variety of malignant tumors of ovarian, liver, pancreas, colorectal, and breast cancer [9,13,14,15,26]. Recently, Changying et al. (2014) [38] demonstrated a higher protein urinary expression of Apo-A1, and found a differential increase in the expression of bladder cancer compared with the healthy controls. All of these findings were further confirmed by Western blot, which further substantiates that a high expression of urine Apo-A1 may be a potential biomarker for bladder cancer. The discrepancies among these reports and our study may possibly be as a result of the types of different tumors investigated, coupled with the different types of races and techniques involved therein.

Non-smokers presented more with higher expression levels with nearly a significant association (*p* = 0.09). In this context, Saha et al. (1994) conducted a study on healthy individuals, and found a higher expression of serum *APOA1* protein in non-smokers [17], which is in contrast with our study performed on bladder cancer, wherein the same group presented more with a lower expression. This finding also points to the fact that smoking leads to urinary bladder inflammation and this may result ina reduction in *APOA1* urinary protein levels. As the current study is the first of its kind, the results obtained on smoking status are difficult to compare due to the lack of other reports.

Among the differential expression of the *APOA1* urinary protein according to gender, a significant association was observed between the males and females (*p* = 0.02). A higher protein expression (≥20 ng) was observed in females, while as it was vice versa for the lower protein expression. This result clearly indicates the differential pattern of *APOA1* urinary protein between gender, but needs to be authenticated in a large cohort series of bladder tumors, as the sample size in our study was less in females (5:1)

For the classification of different *APOA1* levels with the genotypes −75 G/A and 83 C/T, a significant association was observed with respect to variant −75 G/A, with the AA genotype, which had a higher frequency with respect to the elevated expression of the *APOA1* urinary protein (*p* = 0.03). Consequently, the frequency of heterozygotes GA and wild type GG were higher for the lower *APOA1* expression, but were not associated with either higher or lower cut off values. Finally, our results show variant −75 G/A, with the AA genotype, was significantly associated, with a nearly four-fold risk for bladder tumor, as discussed earlier, in addition to its significant relation with *APOA1* urinary expression. The current finding depicts that *APOA1* −75 G/A and its protein expression could act as a promising marker risk assessment and for the development of bladder tumors. However, the frequency of *APOA1* +83 C/T among different cut off values of its urinary protein were randomly distributed, and did not show any relation with any genotype.

No single parameter showed any significant association when the differential expression of the urinary *APOA1* protein was analyzed with respect to the different frequency patterns of *APOA1* −75 G/A and 83 C/T. This shows that the overall *APOA1* concentration and its genotypes have an individual significance with respect to bladder tumors, other than its characteristics.

## 5. Conclusions

The *APOA1* gene −75 AA variant, in concordance with its corresponding relation to the urinary *APOA1* protein expression, revealed its possible role as a marker for the risk assessment of disease in bladder cancer. The *APOA1* combined haplotypic effect confers a significant risk for bladder tumors. Furthermore, the *APOA1* protein expression was concluded to have a possible role as a diagnostic marker for different grades of malignant bladder tumors. As this is the first study of its kind, our results need to be further investigated in a large series of samples in order to authenticate the status of the *APOA*-1 gene and its product, as substantiated in our report. Moreover, our study gives evidence of the vital role of *APOA*-1 in carcinogenesis at genetic and protein levels, and augments further functional studies that can provide additional inputs in the patho-physiological course on different cancers, in particular bladder cancer.

## Figures and Tables

**Table 1:** Distribution of various combinations of genotypes in *APOA1* −75 G>A and 83 C>T (bladder cancer cases and healthy controls).

Genotyp	Cases (%) (*n* = 108)	Controls (%) (*n* = 150)	O.R (95% C.I)	*p* Value *
−75 GG	48 (44.4)	81 (54)	Ref.	
−75 GA	40 (37.0)	60 (40)	1.2 (0.6–1.9)	0.5
−75 AA	20 (18.5)	09 (06)	3.7 (1.24–10.3)	0.001
GA + AA	60 (55.5)	69 (46)	1.2 (0.56–1.99)	0.5
−75 G allele	136 (62.9)	222 (74.0)	Ref.	
−75 A allele	80 (37.1)	78 (26.0)	1.6 (1.03–2.61)	0.004
+83 CC	44 (40.3)	84 (55.0)	Ref.	
+83 CT	54 (50.0)	48 (33.0)	2.1 (1.07–3.97)	0.005
+83 TT	10 (9.7)	18 (12.0)	1.0 (0.54–3.8)	0.9
CT + TT	64 (59.7)	66 (45.0)	1.3 (0.98–3.34)	0.19
+83 C allele	142 (66.7)	215 (72.0)	Ref.	
+83 T allele	74 (33.3)	85 (28.0)	1.3 (0.53–1.40)	0.17

GG—wild; GA—heterozygous; AA—homozygous variant; CC—wild; CT—heterozygous; TT—homozygous variant; * *p* value was calculated by the Fisher exact probability test. Ref—reference taken as wild type compared with variant genotypes/allele.

**Table 2:** *APOA1* gene 75 G>A and +83 C>T polymorphic sequence variations in different clinical parameters.

Parameter	Cases *APOA1* −75		Controls		O.R (95% C.I)	*p* Value	Cases *APOA1* +85		Controls		O.R (95% C.I)	*p* Value
	GG	GA + AA	GG	GA + AA			CC	CT + TT	CC	CT + TT		
Age												
<50 years	18	21	26	19	1.5 (0.67–3.70)	0.4	20	21	25	20	1.4 (0.54–3.70)	0.4
≥50 years	30	39	55	50	1.4 (0.66–3.09)	0.4	28	39	56	49	1.5 (0.71–3.48)	0.3
Sex												
Male	40	50	69	54	1.3 (0.67–2.66)	0.4	42	48	61	60	1.2 (0.61–2.34)	0.6
Female	8	10	12	15	1.6 (0.41–6.07)	0.7	6	12	20	9	4.4 (1.3–15.4)	0.03
Smoking Status												
Smoker	43	50	61	54	1.3 (0.65–2.47)	0.5	39	54	68	47	1.5 (0.72–3.22)	0.1
Non-Smoker	5	10	20	15	2.4 (0.52–11.10)	0.4	5	10	16	19	1.2 (0.41–3.99)	0.4
Grade												
Low	43	45			Ref		18	18				
High	5	15			3.2 (0.07–1.24)	0.04	14	23			1.6 (0.23–1.55)	0.2
Pathologic Stage												
Ta	16	14			Ref		9	8			Ref	
T1	29	34			0.7 (0.3–1.7)	0.7	17	19			0.3 (0.8–1.5)	0.17
T2	3	12			4.4 (3.03–12.3)	0.05	5	13			0.6 (0.22–1.89)	0.2

For *APOA1* −75, GG is taken as the reference against variant GA + AA.

**Table 3:** Haplotype analysis of *APOA1* −75 G>A and +83 C>T for overall association with bladder tumor cases and healthy controls.

Haplotypes		Cases 108 (%)	Controls 150 (%)	OR (95%CI)	*p* Value *
*APOA1* −75	*APOA1* +83				
G	C	74 (34.2)	82 (27.4)	1(Ref)	
G	T	6 (2.8)	28 (9.4)	0.2 (0.06–0.94)	0.03
A	C	4 (1.9)	10 (3.4)	0.4 (0.08–2.5)	0.4
A	T	10 (4.6)	2 (0.07)	5.5 (0.6–49.3)	0.2

* *p* value calculated by Fisher’s exact probability test.

**Table 4:** Urinary protein expression of *APOA1* with respect to clinic o-pathological features and *APOA1* genotypes in bladder cancer patients.

Genotype	Cases (%) APO Expression		O.R (95% C.I)	*p* Value *
	<20 ng/mL	≥20 ng/mL		
*APOA1*				
−75 GG	29 (60.4)	19 (39.5)	Ref	
−75 GA	22 (63.0)	18 (37.0)	0.8 (0.30–2.64)	0.9
−75 AA	6 (30.0)	14 (70.0)	1.5 (0.34–4.59)	0.03
*APOA1*				
+83 CC	30 (65.5)	14 (34.5)	Ref	
+83 CT	30 (44.5)	24 (55.5)	0.6 (0.66–5.24)	0.2
+83 TT	05 (57.1)	05 (42.9)	0.5 (0.54–16.07)	0.5
Grade				
Low	42 (47.5)	46 (52.5)	Ref	
High	17 (84.6)	3 (15.4)	6.08 (1.24–29.8)	0.002
Stage				
Ta	15 (47.6)	15 (52.4)	Ref	
T1	33 (52.3)	30 (47.7)	(0.34–2.81)	1
T2	11 (73.3)	4 (26.7)	2.7 (0.71–10.6)	0.2
Smoking Status				
Yes	54 (58.0)	39 (41.9)	Ref	
No	10 (66.7)	5 (33.3)	1.4 (0.46–4.5)	0.5
Gender				
Male	54 (50.0)	36 (33.4)	Ref	
Female	5 (25.0)	13 (75.0)	4.5 (1.10–18.34)	0.02
Age				
≥50	37 (49.4)	38 (50.6)	Ref	
<50	21 (63.6)	12 (36.4)	0.5 (0.2–1.60)	0.2

* *p* value calculated by Fisher’s exact probability test.

## Data Availability

The data presented in this study are available on request from the corresponding author.

## Supplementary Materials

The following are available online at https://www.mdpi.com/article/10.3390/biomedicines9080938/s1, Table S1: Demographic and Pathological features of bladder cancer cases and controls, Table S2: APOA1 gene expression with respect to different clinical parameters.
