# Supplementary material for: Significant Implications of APOA1 Gene Sequence Variations and Its Protein Expression in Bladder Cancer
Source: Biomedicines. 2021 Aug 2;9(8):938. doi: 10.3390/biomedicines9080938 (PMC8392831; doi:10.3390/biomedicines9080938)
Supplement: Supplementary file 1 [file biomedicines-09-00938-s001.zip › biomedicines-1316243-supplementary.pdf]

Table S1. The primer sequences of Y micro deletions and their interpretation

STS	Locus	Product Size (bp)	Tube	Primer Sequence
SY84	DYS273	326	A	F 5′-AGA AGG GTC TGA AAG CAG GT- 3′<br>R 5′-GCC TAC TAC CTG GAG GCT TC-3′
SY127	DYS218	274	A	F 5′-GGC TCA CAA ACG AAA AGA AA-3′<br>R 5′-CTG CAG GCA GTA ATA AGG GA-3′
SY254	DAZ	380	A	F 5′- GGG TGT TAC CAG AAG GCA AA-3′<br>R 5′- GAA CCG TAT CTA CCA AAG ACG C-3′
SY86	DYS148	320	B	F 5′-GTG ACA CAC AGA CTA TGC TTC- 3′<br>R 5′-ACA CAC AGA GGG ACA ACC CT-3′
SY134	DYS224	301	B	F 5′-GTC TGC CTC ACC ATA AAA CG-3′<br>R 5′- ACC ACT GCC AAA ACT TTC AA-3′
SY255	DAZ	124	B	F 5′- GTT ACA GGA TTC GGC GTG AT-3′<br>R 5′- CTC GTC ATG TGC AGC CAC-3′
SRY		472	A/B	F 5′- GAA TT TCC CGC TCT CCG GA-3′<br>R 5′- GCT GGT GCT CCA TTC TTG AG-3

Table S2. Clinico-pathological features of patients with recurrent miscarriages

Parameters		Total No. 329		Frequency
Age Group	Female	190	<30y	89 (46.8)
			≥30y	101 (53.1)
	Male	190	<30y	50 (26.3)
			≥30y	140 (73.6)
Family History	Female	190	Yes	35 (18.4)
			No	155 (81.5)
	Male	190	Yes<br>No	40 (21.0)<br>150 (79.0)
Consanguinity		190	CM	44 (23.1)
			NCM	146 (76.84)
No. of Miscarriages		190	2	77 (40.5)
			3	75 (39.4)
			4	30 (15.7)
			5	5 (2.6)
			6	2 (1.0)
			8	1 (0.52)
Dwelling		190	Rural	105 (55.2)
			Urban	85 (44.8)
TORCH		140	Positive	4 (2.85)
			Negative	137 (97.14)
APLA		68	Positive	3 (4.41)

		Negative	66 (95.58)
VDRL	60	Positive	0 (0)

Table S3. Various polymorphic changes seen in couples with Recurrent Miscarriages

S. No	Variation	Events	Gender	Frequency (%)
1	46, XX (15p-sat)	3	F	1 (6.7)
2	46, XY,13P-SAT	2	M	1(6.7)
3	46, XX,22p- sat	2/3	F	2(13.3)
4	46, XY 22p- sat	3	M	1(6.7)
5	46, XY,9(qh+)	3/2	M	2 (13.3)
6	46, XY, Y(qh+)	3/5	M	4 (26.7)
7	46, XX, 9(qh+)	5	F	2(13.3)
8	46, XX,14ps+	2	F	1(6.7)
9	46, XY,1qh+	6	M	1(6.7)
Total				15
